# Increasing precision beyond A/T/N:Identification of molecular subtypes of Alzheimer's disease using CSF Proteomics

**DOI:** 10.1002/alz70856_102872

**Published:** 2025-12-25

**Authors:** Bart Smets, Asif Emon, Yanfei Zhang, Jingyue Xi, Liping Hou, Pablo García‐González, Raquel Puerta, Amanda Cano, Alfredo Cabrera Socorro, Shuwei Li, Agustin Ruiz, Christopher D Whelan

**Affiliations:** ^1^ Janssen Pharmaceutica NV, a Johnson & Johnson company, Beerse, Belgium; ^2^ Johnson & Johnson Innovative Medicine, Germany, Wuppertal, Wuppertal, Germany; ^3^ Johnson & Johnson Innovative Medicine, Spring House, PA, USA; ^4^ Janssen Research & Development, LLC, a Johnson & Johnson company, Spring House, PA, USA; ^5^ Ace Alzheimer Center Barcelona – International University of Catalunya (UIC), Barcelona, Spain; ^6^ Ace Alzheimer Center Barcelona, Barcelona, Spain; ^7^ Janssen Research & Development, A Division of Janssen Pharmaceutica, Beerse, Belgium, Beerse, Belgium; ^8^ Glenn Biggs Institute for Alzheimer's & Neurodegenerative Diseases, University of Texas Health Science Center, San Antonio, TX, USA; ^9^ Janssen Research & Development, LLC, a Johnson & Johnson company, Boston, MA, USA

## Abstract

**Background:**

The A/T/(N) framework provides a biological classification for Alzheimer's disease (AD) based on the presence of amyloid plaques (A) and tau protein tangles (T). Fluid and imaging biomarkers for these core AD pathologies enable AD diagnosis even before the onset of clinical symptoms. This has unlocked biomarker‐driven precision neuroscience in research and clinical trials. Yet, the A/T/(N) framework does not capture all biological processes that are involved in AD pathogenesis. Patients often also have co‐pathologies which influence disease progression and response to treatment.

**Method:**

We analyzed CSF proteomics data from 705 individuals with abnormal CSF amyloid levels in the Spanish ACE cohort. ConsensusClustering was applied to study the molecular heterogeneity and identify AD patient subgroups. Clinical, genetic and molecular profiles of these subtypes were compared. XGBoost classifiers were trained to predict CSF subtypes based on plasma proteomics.

**Result:**

Our study identifies 2 robust CSF subtypes: (1) a neuronal hyperplasticity subtype characterized by proteins involved in neurogenesis, axon development and synapse assembly, with elevated CSF PTau‐181 and TTau levels; (2) a blood‐brain‐barrier dysfunction subtype with increased levels of proteins linked to complement, inflammation and coagulation signaling. No differences are observed in neuronal damage markers, rate of MCI‐to‐dementia progression and clinical stage of disease, indicating that the clusters reflect molecular heterogeneity rather than disease stage. Genetic analyses of the subtypes point to differences in underlying disease aetiology and the need for subtype‐specific treatment paradigms. ML models enable accurate prediction of the CSF subtypes from plasma proteomics (AUC > 0.8) and nominate candidate biomarkers.

**Conclusion:**

This study confirms the existence of distinct molecular subtypes of AD that stratify patients beyond A/T/N, corroborating findings by Tijms et al. (2024) & Moutinho et al. (2024). Further validation of these subtypes in additional cohorts and clinical trials, alongside the development of blood based biomarkers, will help to further enhance the precision of AD diagnosis, prognosis, and treatment.

